# A Unified Probabilistic Framework for Dose–Response Assessment of Human Health Effects

**DOI:** 10.1289/ehp.1409385

**Published:** 2015-05-22

**Authors:** Weihsueh A. Chiu, Wout Slob

**Affiliations:** 1 National Center for Environmental Assessment, Office of Research and Development, U.S. Environmental Protection Agency, Washington, DC, USA; 2 National Institute of Public Health and the Environment (RIVM), Bilthoven, the Netherlands

## Abstract

**Background:**

When chemical health hazards have been identified, probabilistic dose–response assessment (“hazard characterization”) quantifies uncertainty and/or variability in toxicity as a function of human exposure. Existing probabilistic approaches differ for different types of endpoints or modes-of-action, lacking a unifying framework.

**Objectives:**

We developed a unified framework for probabilistic dose–response assessment.

**Methods:**

We established a framework based on four principles: *a*) individual and population dose responses are distinct; *b*) dose–response relationships for all (including quantal) endpoints can be recast as relating to an underlying continuous measure of response at the individual level; *c*) for effects relevant to humans, “effect metrics” can be specified to define “toxicologically equivalent” sizes for this underlying individual response; and *d*) dose–response assessment requires making adjustments and accounting for uncertainty and variability. We then derived a step-by-step probabilistic approach for dose–response assessment of animal toxicology data similar to how nonprobabilistic reference doses are derived, illustrating the approach with example non-cancer and cancer datasets.

**Results:**

Probabilistically derived exposure limits are based on estimating a “target human dose” (*HD_M_^I^*), which requires risk management–informed choices for the magnitude (*M*) of individual effect being protected against, the remaining incidence (*I*) of individuals with effects ≥ *M* in the population, and the percent confidence. In the example datasets, probabilistically derived 90% confidence intervals for *HD_M_^I^* values span a 40- to 60-fold range, where *I* = 1% of the population experiences ≥ *M* = 1%–10% effect sizes.

**Conclusions:**

Although some implementation challenges remain, this unified probabilistic framework can provide substantially more complete and transparent characterization of chemical hazards and support better-informed risk management decisions.

**Citation:**

Chiu WA, Slob W. 2015. A unified probabilistic framework for dose–response assessment of human health effects. Environ Health Perspect 123:1241–1254; http://dx.doi.org/10.1289/ehp.1409385

## Introduction

The process of identifying human health hazards of chemicals has evolved substantially over time with advances in weight of evidence determination, mode of action (MOA), and systematic review (e.g., Meek et al. 2014; NRC 2011; U.S. EPA 2005; Woodruff and Sutton 2014), but practices for quantitative dose–response assessment to characterize those hazards and inform risk management rely largely on approaches that have shown relatively small changes since they were first used. For assessment of non-cancer effects, it is still common to derive exposure limits by dividing a no observed adverse effect level (NOAEL) or a benchmark dose lower confidence limit (BMDL) derived from a chronic study by a generic “uncertainty factor” of 100 (also known as a “safety,” “assessment,” or “extrapolation” factor), although using chemical-specific “adjustment” factors (CSAFs) or data-derived extrapolation factors (DDEFs) is increasingly encouraged (IPCS 2005; U.S. EPA 2014b). Exposure limits for carcinogens that are genotoxic or without an established nongenotoxic MOA are usually based on other approaches, in particular the linear extrapolation approach (EFSA 2005; U.S. EPA 2005), although more recently the margin-of-exposure approach has been suggested even for genotoxic carcinogens (Barlow et al. 2006; Benford et al. 2010; O’Brien et al. 2006).

Although procedurally straight-forward, these practices are most amenable to risk management decisions in which the margins between calculated exposure limits and actual or anticipated exposures are large enough to be of little or no risk management concern. For instance, the safety factor approach results in an exposure limit [acceptable daily intake (ADI), reference dose (RfD)] generally presumed to be “safe” (e.g., having “reasonable certainty of no harm”). However, the conclusion that exposures at or below this level would not result in appreciable health risks is typically not based on further quantitative substantiation. For exposures higher than such an exposure limit, the only statement that can be made is that risks “cannot be excluded” without any quantitative characterization of what the extent of potential health effects might be. Thus, if reducing exposures to the level of the derived exposure limit is challenging (e.g., economically, practically, or politically), then there is no way to weigh the cost of exposure reduction against its likely human health benefits. Moreover, there may be a residual risk at, or even below, the exposure limit, and this residual risk may vary among different chemicals and/or exposure scenarios.

To address these disadvantages, a probabilistic approach to hazard or risk characterization has been advocated by several risk assessment researchers (Baird et al. 1996; Evans et al. 2001; Hattis et al. 2002; Slob and Pieters 1998; Swartout et al. 1998), as well as by several expert panels (NRC 1994, 2009; U.S. EPA Science Advisory Board 2002). Although most of the work has focused on characterization of non-cancer effects, the National Research Council (NRC) revisited the question of unifying assessment of cancer and non-cancer effects (NRC 2009). Some of the recommendations of NRC (2009) as to default approaches to low-dose extrapolation have been controversial (Abt et al. 2010; Pottenger et al. 2011; Rhomberg 2011; Rhomberg et al. 2011); however, deciding on such science policy questions (as “default” options clearly are) does not preclude moving forward with developing a unified probabilistic framework for all types of endpoints.

In this review we present a unified probabilistic framework, developed in tandem with an international harmonization project (IPCS 2014) on uncertainty in human dose–response assessment [“hazard characterization” in World Health Organization/IPCS (International Program on Chemical Safety) nomenclature]. This framework retains the usual “two-part” process as employed in the current nonprobabilistic (“deterministic”) approaches for quantitative dose–response assessment: *a*) dose–response analysis of an experimental or observational dataset of health effects resulting from chemical exposure, and *b*) inference (or “extrapolation”) as to the potential effects in the target human population. The second part needs to account for differences in characteristics (e.g., species, exposure duration) between the dataset analyzed and the human population of interest for risk assessment. In terms of the usual deterministic approach of dose–response assessment, the determination of the point of departure (POD) may be regarded as the first part and the extrapolations addressed by uncertainty factors (interspecies, intraspecies, subchronic-to-chronic, etc.) as the second part. Although the basic procedure appears unchanged, probabilistic assessment requires more precise definitions of each step and thus also promotes greater transparency as to the biological and quantitative assumptions underlying the dose–response assessment. In particular, the framework we propose here provides a theoretical basis for human dose–response assessment, where all underlying concepts are explicitly defined and logically interrelated. In this way, it is fully transparent as to what the various computational procedures represent, and how the results can be interpreted.

This review is organized as follows. In “Methods,” we first set out the four fundamental principles that underlie the unified probabilistic framework. In addition, we lay out a prototypical approach to implement the unified framework for human-relevant animal toxicology data. In “Results,” we illustrate the approach by deriving probabilistic exposure limits from example non-cancer and cancer datasets using probability distributions for uncertainty derived from historical data (for datasets and computer code, see Supplemental Material, Table S1). In “Conclusions,” we discuss implementation issues and identify data needs (see also Supplemental Material, “Additional Applications and Extensions”).

## Methods

*Fundamental principles*. Principle 1. Individual and population-level dose response. The starting point of this framework is that a conceptual distinction exists between effects on the individual and effects on the population. In particular, the effect of exposure at the level of the individual is the “magnitude” of a measure of toxicological effect. The result of a fixed exposure in a population will be different magnitudes of effect in different individuals in that population. Therefore, for a particular magnitude of effect, the result in the population is expressed as an “incidence.” In the present framework, the magnitude of change needs to be ordinally related to severity—so a greater magnitude constitutes a more severe effect. For instance, a body weight (BW) decrease of 20% is more severe than a BW decrease of 10%, and a moderate liver lesion is more severe than a mild liver lesion. Thus, for a monotonic dose response in an individual, it may be imagined that a higher exposure will, for any given endpoint, lead to more severe effects. In a population, increasing exposure levels will result in simultaneously increasing both incidence and severity: more and more individuals will suffer from more and more severe effects.

For convenience, we establish the notation whereby human dose or exposure is denoted *HD*, the magnitude of effect is denoted by *M*, and incidence is denoted *I*. Because *M* is assumed to have an ordinal relationship with severity, incidence can be characterized as the incidence of effects of magnitude equal or greater than *M*, denoted *I*_≥M_. Because it is customary to discuss “incidence” in terms of effects that may be of concern, we use the simpler notation *HD_M_^I^* as shorthand for *HD*(*I*_≥M_). In addition, we use an asterisk (*) to indicate fixed or target values, such as a “critical effect size” (M*), target human dose (HD*), or target incidence level (I*).

Given these definitions, the output of a human dose–response assessment is concerned with the quantitative relationships among *HD*, *M*, and *I*, along with their uncertainty. We focus on the most common type of output, which is developing a human health–based exposure limit (some other types of outputs are discussed in Supplemental Material, “Performing a population assessment”). In our notation, this means estimating a target human dose, HD*, which is regarded as a function of a two-dimensional protection goal: the target level of incidence (I*) and the specified level of effect magnitude (M*), both of which may be selected based on risk management considerations. Specifically, it is the dose at which only a small fraction of the population (low incidence of I*) will experience effects ≥ M*, which can be written

HD* = *HD*(I*_≥M*_) = HD_M*_^I*^. [1]

For instance, one could write *HD*(1%_≥ 10%BW_) = *HD*_10_^01^ for the dose at which only 1% of the population has > 10% change in BW.

Principle 2. Continuous parameters underlying all observed dose–response endpoints. The second element of this framework is that observed dose–response relationships for all endpoints can, at the individual level, be recast as relating to an underlying continuous measure of response. Obviously, this principle applies to endpoints that are directly observed as continuous data. In that case, the observed dose response for the average responses as a function of dose may be imagined to reflect the dose response in an individual animal (namely, the average animal), even though an individual’s dose response is not directly observable in most toxicological studies. Endpoints that are generally measured as quantal response rates in a study population require some additional discussion. Below we discuss two options, briefly indicated as “deterministic” and as “stochastic” quantal endpoints (Slob et al. 2014).

**Figure 1:**
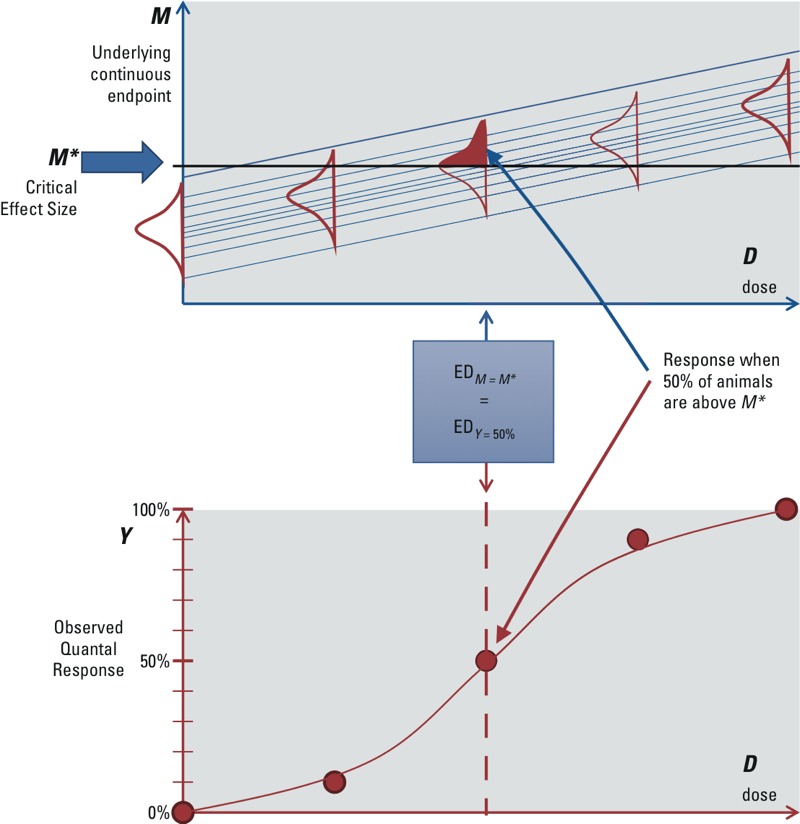
Deterministic quantal endpoints: quantal responses reflecting incidences of a continuous response above/below a fixed cut point. The various dose–response lines in the upper panel reflect the (hypothetical) dose responses of individual animals.

Many quantal endpoints, such as in histopathology, are ordinally scored (e.g., minimal, mild, moderate, severe). Such endpoints can be considered as gradually increasing in magnitude at the individual level, but are reported in “bins,” or severity categories rather than as a continuous measure. In this way, the reported incidences can be thought of as relating to a single category (or a limited number of categories) of severity, whereas for other severities the incidences are not reported. In fact, any continuous dataset can be “quantized” and transformed into such a quantal (or ordinal) dataset by setting one (or more) cut points, resulting in incidences *Y* associated with each cut point. For instance, changes in hematocrit from the mean level in the controls can be separated into < 5% and ≥ 5%, and the fraction of animals below and above the 5% cut point can treated as quantal data. In this case, the effective dose (ED) for a 5% change in the continuous hematocrit data (i.e., ED_05_) is equal to the ED for a 50% quantal response, ED*_Y_*_= 50%_, as illustrated in Figure 1. This concept of quantal endpoints has previously been discussed by Slob and Pieters (1998).

Therefore, when quantal data can be viewed as reflecting the incidence of a continuous effect above or below a “determined” cut point equal to M*, then the endpoint is referred to as a “deterministic quantal endpoint.” This is generally appropriate for effects that can occur in different degrees of severity. Furthermore, for the purposes of dose–response data analysis, the ED_50_ from the quantal response data would be used to estimate the ED_M__*_ corresponding to M* of the underlying continuous data. When the available dose–response data report the incidences related to various severity categories, then one of them may be chosen as being minimally adverse. When they report only the incidences related to a single severity category, this severity may be more than minimally adverse, in which case additional uncertainty arises in estimating the dose for a minimally adverse level of severity.

**Figure 2:**
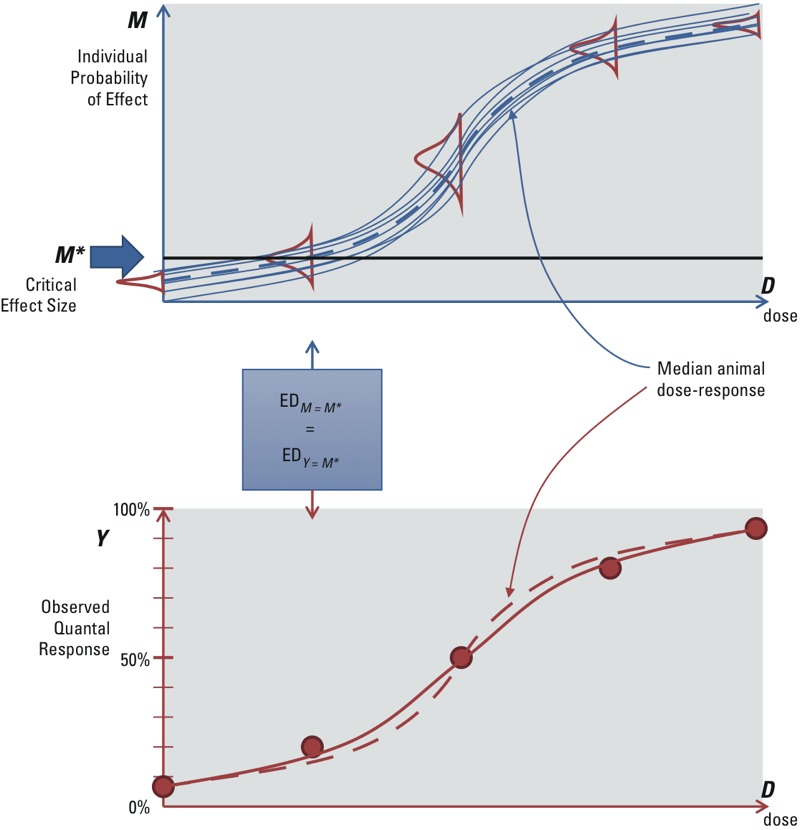
Stochastic quantal endpoints: quantal responses reflecting individual probability of effect. The dashed lines in the upper and lower panel are the same, representing the (hypothetical) dose response of the median animal. In the upper panel, the solid lines represent (hypothetical) individual dose–response curves. In the lower panel, the solid line reflects the expected value of the observed quantal response from the population of individual responses, which is less steep than the dose response of the median animal.

However, not all quantal effects may be derived from applying a cut point to an underlying continuous variable. Some effects appear to have discrete outcomes, without an underlying, gradually increasing level of severity. An example of such an endpoint is malformations, which often do not show different degrees of severity: It is there or it is not. Cancer may be considered another example, because a particular tumor is present or not (ignoring observational practicalities). For such endpoints, an alternative interpretation of the dose response is possible: The observed incidences at each dose are considered as resulting from a “stochastic” process, where the observation that an individual animal has a tumor or not is analogous to drawing a lottery ticket, with probability equal to the expected incidence at that dose (and time of observation). That is, given all relevant circumstances for the particular individual (such as genetic make-up or experimental conditions), the effect is not fully determined, but rather any particular animal may be (un)lucky or not. If it were possible to perform a study in which all animals were identical, and identically treated (except the dose, but without dosing errors), then the quantal dose response would estimate the “individual probability of effect.” In this case, the observed incidence *Y* is treated as an estimate of the underlying individual probability of effect *M*, so M* would correspond to an incidence Y* = M*, as depicted in Figure 2. This concept of quantal endpoints has previously been discussed by Slob et al. (2014).

Therefore, when quantal data are assumed to reflect the individual probability of an outcome as a result of a stochastic process, the endpoint is referred to as a “stochastic quantal endpoint.” In reality, there are always small differences between animals (including the experimental conditions), which will have some impact on the dose response. However, this additional impact is generally not separately distinguishable from the dose–response data. This interindividual variability might be assumed to be relatively small in the study population and hence ignored. If so, the observed quantal dose response approximates the individual probability of effect as a function of dose.

Whether the “deterministic” or “stochastic” interpretation of quantal endpoints is correct remains uncertain, because even for endpoints such as cancer or malformations, it might be the case that the effect in an individual subject is evoked deterministically as soon as a given internal dose in that individual is reached. For risk assessment, the distinction between the two interpretations is important for the following reason: In the deterministic interpretation, the animal dose–response curve reflects the experimental variation and errors in the animal study, and therefore its shape (e.g., slope) would not be relevant information for predicting risks in humans. However, in the stochastic interpretation, the dose–response curve may be regarded as a model for the human individual probability of effect, and therefore its shape would be relevant as information for human risks.

Principle 3. Selecting a basis for inference: the “effect metric.” The third fundamental element in this framework is that inferences are made on the basis of a selected “effect metric*”* that defines “toxicological equivalent” magnitudes of effect. This effect metric should reflect the effect size in such a way that it applies across species (or populations) as well as across individuals within a species (or population). Changes of the same magnitude in this metric are considered to reflect equal toxicologically induced changes.

In addition, the magnitude of effect should also be ordinally related to severity at the level of the individual. For a continuous endpoint, severity increases with an increase in the percent change of a continuous endpoint (e.g., from 5 to 7% decrease in hematocrit). For a deterministic quantal endpoint, the severity is related to the category of effect (e.g., from “mild” to more severe liver lesions). For a stochastic quantal endpoint, severity is related to the probability of experiencing the effect (e.g., from 1 to 2% individual probability of cancer).

“Equipotent doses” are defined as doses that elicit the same size of effect metric. Thus, individuals with the same equipotent doses (at all effect sizes) are defined as equally sensitive to the chemical for the endpoint.

Note that it is assumed that the effect has previously been determined to be an appropriate basis for making inferences about human health effects—for example, that the effects observed in the test animal are relevant to humans. In this context, “relevance” needs to be determined only in the qualitative sense: Could a similar effect occur in humans, or not? When the answer is “yes,” quantitative differences, including large differences that are expected based on MOA considerations, should be addressed explicitly and quantitatively, taking uncertainty into account as well (e.g., using a probabilistic CSAF or DDEF; see Supplemental Material, “Chemical-specific/data-derived toxicokinetics or toxicodynamics”).

The use of an effect metric does not necessarily imply that a given change is equally adverse in all individuals (or species). For instance, a 5% decrease in hematocrit may be considered as a toxicologically equivalent effect metric in all individuals, but be adverse in persons with anemia and nonadverse in healthy persons. Finally, it should be noted that further inferences are possible from the toxicologically equivalent effect metric to other measures of health effect, such as if an adverse outcome pathway can quantify the linkage between a change in effect metric and the likelihood of an adverse health outcome. For instance, if the effect metric is a percent change in serum cholesterol, given an adverse outcome pathway linking serum cholesterol changes to cardiovascular disease, one might aim to estimate the risk of fatal myocardial infarction in a specific population. Because variability in baseline serum cholesterol levels and other relevant risk factors (e.g., blood pressure, C-reactive protein) may differ across different populations (e.g., geographic regions, socioeconomic groups, lifestages), analyses of such “downstream effects” would necessarily be specific to the population(s) being assessed, even if the relationship between exposure and the effect metric is assumed to be the same across populations. Such analyses may also be useful for socioeconomic analyses because a fixed magnitude of effect may have different cost implications across human subpopulations (e.g., modifying insulin for diabetics vs. nondiabetics). This is discussed further in Supplemental Material, “Extrapolation to downstream health endpoints and adverse outcome pathways” and Figure S1.

Principle 4. Making adjustments while accounting for variability and uncertainty. The final fundamental element in this framework is that dose–response assessment involves making inferences about the human population of interest for risk assessment (the “target population”) based on information obtained from a scientific study (the “study population”). In the usual deterministic approach, these inferences are accomplished using the “uncertainty factors” to address (potential) differences due to differing species, human variability, suboptimal study conditions, and so on. However, these factors are often mixtures of multiple elements that need to be clearly specified in a probabilistic framework. Specifically, making inferences between the “study” and “target” populations involves making adjustments from the study to the target populations while accounting for variability and uncertainty:

Adjustments are needed to correct for differences between the study and target populations in order to make inferences as to the potential health effects in the population of interest, with the relevant exposure conditions. For example, on average across chemicals, the dose in milligrams per day eliciting the same effect differs between species due to differences in body size. The usual (implicit) adjustment is to divide the dose by BW, which is also intended to normalize across individual subjects in the (study or target) population. But data increasingly support the idea that the dose in milligrams per kilogram BW may need additional adjustment by an allometric scaling factor to achieve equivalent effects (e.g., Bokkers and Slob 2007; Dedrick 1973; Kleiber 1932; Price et al. 2008; U.S. EPA 2011b). Further, it might be known that, for any particular chemical, there are specific differences in toxicokinetic or toxicodynamic properties, which, for instance, make it plausible that one species would be more sensitive than others (e.g., resulting in a CSAF or DDEF). As another example, for some classes of effects, the expected relationship between a benchmark dose (BMD) and duration of exposure might be reflected by Haber’s law (toxicity depends on the product of concentration and exposure time), which may be used to adjust the BMD to other exposure durations. Usually, differences in study population/conditions and the target population/conditions can be better characterized (i.e., its uncertainty reduced) with additional data or analysis, and some can even be eliminated by conducting new studies that require fewer adjustments (e.g., conducting a chronic study to replace a subchronic study).
“Variability” refers to intrinsic heterogeneity about a central tendency, usually between the individuals in the “target” population. For example, different individuals (humans) will exhibit different sensitivity to toxic effects from the same exposure due to various sources of variability (e.g., genetics, lifestyle, health status). Additional data or analysis can make an estimate of human variability more precise, but the variability itself cannot be eliminated.
“Uncertainty” refers to a lack of knowledge that could, in principle, be reduced with additional data or analysis. Uncertainty can relate to the degree of adjustment (e.g., the exact allometric power with which to adjust for BW differences) but also to the degree of variability (e.g., how much more sensitive is the 95% individual relative to the median individual). As another example, because toxicity studies have finite numbers of individuals per dose group, the BMD is uncertain. This uncertainty can, in principle, be reduced by performing a larger or better designed study. Similarly, “missing studies” represent an uncertainty that can be quantified by meta-analyses comparing the overall differences between study types and capture that in a distribution (e.g., Hattis et al. 2002). In some cases, observed variability among chemicals, in general, can be used to inform the uncertainty in an adjustment factor for a specific chemical. For instance, observed variability among chemicals in the dose ratio between subchronic and chronic studies for the same effect translates into uncertainty in the subchronic/chronic difference for a specific chemical for which no such data are available (e.g., Bokkers and Slob 2005).

As Table 1 shows, all typical uncertainty factors include an uncertainty component, and an adjustment component, except for the intraspecies factor, where the adjustment component is replaced by a variability component.

**Table 1:** Components of adjustment, variability, and uncertainty in some typical uncertainty factors.

Uncertainty factor	Adjustment	Variability	Uncertainty	Comment
Correcting dose for body size	✓		✓	Oral dose in mg/day may be adjusted to mg/(kg^α^ day), where the value of α may be chosen to be 1 or < 1; this value is assumed to hold generically, so there is no variability, but the value of α is uncertain. Generic adjustments have also been derived for inhalation exposures based on regional gas or particle dosimetry derived from respiratory tract geometry and airflow.
Interspecies toxicokinetic or toxicodynamic differences	(✓)^*a*^		✓	Assuming that the test animal and humans are (on the appropriate dose scale) equally sensitive, on average, to chemicals overall, no further adjustment is needed (i.e., the factor equals 1). However, species do differ in sensitivity from one chemical to another. This chemical-to-chemical variability translates into uncertainty about the appropriate factor for a single chemical.
Intraspecies		✓	✓	Some humans are expected to be more sensitive than others, but for a single chemical and effect, it is uncertain how many of them are more sensitive and by how much. Thus, there is variability, the size of which is uncertain.
Subchronic/chronic	✓		✓	On average, for chemicals overall, a given effect may be expected to occur at a lower dose with chronic exposure than with subchronic exposure (hence adjustment), but a single chemical may deviate to an uncertain degree.
Database	✓		✓	When one study type systematically results in lower PODs, then adjustment would be needed, while a single chemical may deviate to an uncertain degree.
^***a***^The adjustment factor is assumed to be 1 in this case, so that it appears to be absent in the calculations.				

*Prototypical approach implementing a unified probabilistic framework.* The principles described above underlying a unified probabilistic framework can be applied to any type of study or endpoint that has dose–response information, but here we address the most common case of using animal toxicology data. The primary assumption is that the candidate critical endpoint(s) from an animal toxicology study is relevant in the sense that similar effects might be expected to occur in humans (uncertainty in the qualitative cross-species concordance is not addressed in this framework). Additional assumptions are as follows:

The toxicity data are from a study conducted in an (inbred) laboratory animal strain, with the purpose of mimicking what might happen in a typical human being. Intrastudy variability reflects experimental errors (e.g., dosing errors, imperfectly controlled experimental conditions) and remaining differences (genetic, or otherwise) among animals. This is treated as statistical uncertainty in estimating a POD, which is supposed to mimic an equipotent dose in a typical human being.
In the effect range of interest, the continuous dose–response relationships are monotonic and parallel on a log-dose scale across species and across individuals within a species, so that the values (distributions) for any adjustments, variability, or uncertainties are independent of the selected critical effect size M*. Slob and Setzer (2014) found evidence consistent with this assumption.

**Figure 3:**
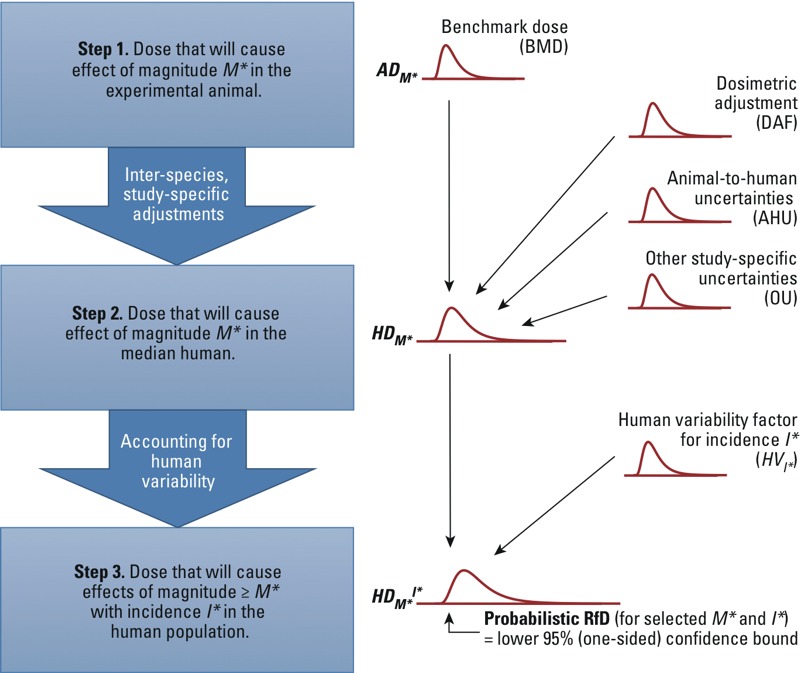
Implementation of the unified probabilistic framework to derive the uncertainty distribution for HD_M*_^I*^ and a corresponding probabilistic RfD. In step 1, BMD analysis is used to derive the uncertainty distribution for AD_M*_. In step 2, this distribution is combined with uncertainties in dosimetric adjustment, animal-to-human toxicokinetics and toxicodynamics, and other study-specific limitations, to derive the uncertainty distribution for HD_M*_. In step 3, the distribution is further combined with the uncertainty in the human variability factor corresponding to the selected incidence I* in the population to derive the uncertainty distribution for HD_M*_^I*^. The lower 95% (one-sided) confidence limit on HD_M*_^I*^ can be chosen as the “probabilistic RfD” corresponding to the selected values of M* and I*. See “Methods” and Table 2 for additional details. This approach is illustrated with two example datasets, with results shown in Table 4 and Figures 4 and 5.

The basic steps of the procedure under these assumptions are as follows (see also Figure 3 and Table 2):

**Table 2:** Summary of unified probabilistic framework.

Step and goal	New input(s) for each step	Output(s) for each step
1. Critical ED in animal. Estimate the uncertainty distribution for *AD*_M__*_, the animal dose associated with the critical effect size M*.	Animal dose–response data Toxicologically equivalent effect metric (*M*) Critical effect size (M*) Appropriate BMD analysis	*AD*_M__*_ = uncertainty distribution for BMD based on analysis of animal dose–response data.
2. Equipotent dose in median human. Infer the uncertainty distribution for *HD*_M*_ = *HD*(0.5_≥M*_), the human dose at which 50% of the human population has effects greater than or equal to the critical effect size M*.	*AD*_M_* from step 1 *DAF*, distribution for the dosimetric adjustment factor due to differences in body size between animal and human *AHU*, distribution for the “animal-to-human uncertainties” due to unknown chemical- and/or species-specific toxicokinetic or toxicodynamic differences *OU*, the distributions for “other uncertainties” due to study- and/or endpoint-specific conditions that differ from the target conditions	*HD*_M__*_ = *AD*_M__*_ × *DAF*/ (*AHU* × *OU*) = uncertainty distribution derived by multiplying *AD*_M__*_ by uncertain factors.
3. Equipotent dose in sensitive human (for an exposure limit). Infer *HD*_M*_^I*^ = *HD*(I*_≥__M*_), the dose at which a target incidence I*_≥__M__*_ yields effects of size ≥ M*. Select a particular value HD* from the uncertainty distribution based on level of confidence.	*HD*(0.5_≥M__*_) from step 2, serving as the uncertainty distribution for the median of the human variability distribution A log-normal human variability distribution, and a separate uncertainty distribution for its variance σ_*H*_^2^^*a*^ A target incidence I*, from which a human variability factor *HV*_I*_ for the ratio between the “sensitive” and median individual is calculated [= exp(*z*_I*_ σ_*H*_) for a log-normal distribution, where *z*_*I*_ is the normal *z*-score for the I* quantile]	*HD*_M__*_^I*^ = *HD*_M__*_ × *HV*_I*_ = uncertainty distribution for the I* percentile of a human variability distribution with median equal to *HD*_M__*_ and standard deviation on log scale of σ_*H*_.
^***a***^We use a log-normal distribution for the uncertainty in the variance, but other distributions could in principle be used.

Select a toxicologically equivalent effect metric and an associated critical effect size (M*), and conduct a BMD analysis with benchmark response (BMR) = M* (Crump 1984) to derive the uncertainty distribution for the dose corresponding to M* in the animal (AD_M__*_).
Apply probabilistic interspecies and other adjustments to AD_M__*_ to derive the uncertainty distribution for the dose corresponding to M* in the median human (HD_M__*_).
Select a human variability distribution (e.g., log-normal), setting the median to HD_M__*_ with an uncertainty distribution as obtained in step 2. The measure of dispersion of this human variability distribution [such as geometric standard deviation; GSD = exp(σ*_H_*)] has an uncertainty distribution, reflecting that we are uncertain about the degree of variability among individuals. From this (uncertain) human variability distribution, we derive an (uncertain) human variability factor HV_I*_ for the ratio between the quantile corresponding to a selected target incidence (I*) value and the median, so that HD_M*_^I^* = HD_M*_ × HV_I*_.

This output is an estimate of the HD_M*_^I^* in the form of an uncertainty distribution, and any given level of confidence may be chosen for deriving an exposure limit (e.g., a “probabilistic RfD”), by taking the associated lower percentile of the uncertainty distribution of HD_M*_^I*^. Alternatively, the full uncertainty distribution can be combined with exposure information to inform risk management decisions. Details of each step are described below along with Monte Carlo (MC) procedures for the overall calculation.

Step 1: Estimating the animal dose corresponding to the critical effect size for the selected toxicologically equivalent effect metric. The purpose of this step is to establish the uncertainty distribution for AD_M*_, the animal dose associated with a specified effect size M* (= BMR) based on a specified toxicologically equivalent effect metric.

The key issue in defining the effect metric is how to address baseline differences across species or individuals in order to make changes “comparable.” For instance, a decrease of 10 g in rat body weight does not compare to a 10-g change in human body weight. For most (continuous) parameters, a percent change would be the obvious effect metric, being the only measure that may be defined as representing an equal effect size among different species and individuals (with different background responses). Note that an equal effect size does not imply that it will always be equally adverse in different species/individuals (such as a 5% decrease in hematocrit in anemic vs. nonanemic persons). Severity categories in histopathological lesions appear to directly apply as a measure of equivalent effect magnitude. However, for endpoints measuring an increase in individual probability of effect, the question of how to correct for the background risk is not easily answered. Various measures are being used, such as additional, extra, or relative risk, which all correct for background in a different way. It remains unclear, however, which of these measures reflects an equivalent measure of risk (if any), in particular when background risks among species (populations) differ greatly.

After having chosen the effect metric, one also needs to specify a critical effect size—the magnitude of effect size M* of interest, as defined by the problem formulation and risk management context for the assessment. The term “critical*”* here should be understood in a wide sense, that is, it is a selected value (or even a range of values) that forms a starting point for doing the probabilistic calculations. Often, the problem formulation suggests that the critical effect size should reflect the effect size that is considered to be “minimally adverse” biologically. However, current toxicological knowledge does not allow one to unequivocally define minimally adverse effect sizes for all potentially critical endpoints. Further, a given effect size might not be minimally adverse in one species or individual, while it is minimally adverse in another (e.g., hematocrit and anemia, discussed above). As a practical limitation, the choice of *M* may be restricted by the available data. For instance, the reported data may relate to discrete values of *M* only (e.g., specific severity categories of lesions, as in histopathological data). Moreover, the lower the value of *M*, the less precise the estimates of the associated doses will be. For biologically defined M*s, one might aim to specify study designs that are likely to achieve “adequate” statistical precision for dose estimates related to that value M*. However, even then, the study design needed to achieve that goal may be impractical (e.g., unrealistic number of animals needed). If so, one may decide to use a statistically based M* (i.e., the lowest value of M* that achieves the desired level of statistical precision) as a surrogate. Such statistically based M*s could reflect levels of effect that are larger than minimally adverse levels, and this can be regarded as an additional source of uncertainty or addressed by setting a more stringent protection goal in terms of incidence. Typical examples of effect metrics and critical effect sizes are shown in Table 3, along with the BMD approach implied, by treating all endpoints as fundamentally continuous.

**Table 3:** Example approaches to analysis of the animal dose–response data.

Endpoint type (examples)	*M*: Example of toxicologically equivalent effect metric	M*: Example of critical effect size(s)	Benchmark dose approach
Continuous (hematocrit, serum enzyme, BW, organ/BW ratio)	Percent change relative to control	5%, 10% (percent change)	Continuous models with BMR = M* = 5%, 10%.
Deterministic quantal (hepatic lesions, cytoxicity)	Severity category	“Minimal” (severity category)	Quantal models for 50% incidence of M* = minimal, mild.
Stochastic quantal (hepatic tumors, fetal resorptions, eye malformations)	Extra risk for individual probability of occurrence	1%, 5%, 10% (extra risk)	Quantal models with BMR = M* = 1%, 5%, 10%.

The result of the dose–response analysis is an uncertainty distribution for AD_M__*_, the animal dose corresponding to M*. Approaches to establishing the uncertainty distribution include *a*) translating the BMD confidence limits obtained by BMD software into a distribution, *b*) parametric bootstrapping [Slob and Pieters 1998; implemented in the R package (version 3.2.2; R Core Team 2015) PROAST (RIVM 2012)], or *c*) Bayesian analysis (Kopylev et al. 2007). It should be noted that fitting a single dose–response model may not fully capture the uncertainties in the dose–response data. Therefore, instead of deriving a BMD distribution from a single model, various models should be fitted to address model uncertainty. These model-specific distributions may be simply pooled in a single distribution (e.g., Slob et al. 2014), or one may apply “model averaging,” for which various approaches have been proposed (Bailer et al. 2005; Shao and Gift 2013; Wheeler and Bailer 2007). In addition, if different dose–response datasets are available for the same endpoint, they could be combined in a joint dose–response analysis, with study as a covariate in the analysis, that is, some of the parameters of the dose–response model are study specific, and others are not (Slob and Setzer 2014).

Step 2: Adjustments due to interspecies differences and study conditions. The purpose of this step is to establish an uncertainty distribution for the “typical” human dose associated with a specified magnitude of effect and endpoint, and with specified exposure conditions. This step combines with the results of step 1. The “typical” human is defined as the median person of the population. This interspecies step involves addressing three separate aspects:

A dosimetric adjustment factor (DAF) for generic physiological differences (e.g., body size differences for oral dose; respiratory tract differences for inhalation exposures) between the test animal and (median) human, along with uncertainty in the expected adjustment
Animal-to-human uncertainties (AHU) due to potential chemical-specific toxicokinetic or toxicodynamic differences between the test animal and humans, resulting in differences in sensitivity for a given chemical
Other uncertainties (OU) due to specific study conditions that differ from the target exposure conditions (e.g., exposure duration, or exposure pattern).

The result of this step is an uncertainty distribution for the human dose at which 50% of the human population has effects greater than (or equal to) M*:

*HD*(0.5_≥M__*_) = AD_M*_ × *DAF* / (*AHU* × *OU*). [2]

Each of the adjustments is described in more detail below.

*Dosimetric adjustments.* It is increasingly evident that generic differences in physiology (e.g., body size) across species can be accounted for by multiplying the animal dose by a DAF, or equivalently, by dividing by an “assessment” factor (AF) accounting for interspecies body size differences (*AF*_inter-bs_).

For oral exposures, scaling doses by a fractional power of BW has been found to better account for interspecies differences in body size than scaling by BW alone. Because oral doses are usually expressed as milligrams per kilogram BW, a correction factor is needed to convert the doses in milligrams per kilogram into allometrically scaled doses. Thus, the *DAF* and *AF*_inter-bs_ are given by

*DAF*_oral_ = (animal BW/human BW)^1 – α^ [3]

*AF*_inter-bs(oral)_ = (human BW/animal BW)^1 – α^, [4]

where α is the allometric power. This power is not exactly known, and can be represented by a distribution (e.g., normal). Because this adjustment is meant to extrapolate from the test animal to the median human, the average (median) animal BW in the study and the median human BW in the target (sub)population should ideally be used (U.S. EPA 2011a). If these are not available, then standard values can be used (e.g., U.S. EPA 1988), with an uncertainty that is probably negligible compared with the uncertainty in the allometric power (although the BW uncertainty could be included in the assessment).

For inhalation exposures, different types of DAFs have been derived for particles (regional deposited dose ratio, or RDDR) and gases (regional gas dose ratio, or RGDR) (U.S. EPA 1994). Based on interspecies information about respiratory tract geometries and air flow rates, the inhalation DAFs differ depending on whether the effects of interest are regional or systemic. For example, for effects in the upper airways, DAFs are based on the surface areas of relevant regions of the respiratory tract and the inhalation minute-volume. For systemic effects that involve transport by blood, DAFs utilize information on species differences (if any) in blood-air and blood-tissue partition coefficients. As with the oral DAFs, these are meant to extrapolate between the (median) test animal and the median human. Standard values, rather than statistically based medians or values specific to the study, are usually employed, but clearly these are uncertain as well. Thus, one could define the uncertainty in the DAF (or in the analogous *AF*_inter-bs(inhalation)_) by assuming log-normal residual uncertainty:

*DAF*_inhalation_ = (RDDR or RGDR) × exp(ε*_DAF_*) [5]

*AF*_inter-bs(inhalation)_ = (RDDR or RGDR)^–1^ × exp(ε*_DAF_*), [6]

where ε*_DAF_* is normally distributed with a standard deviation of σ*_DAF_*. The value of σ*_DAF_* might be based on propagating the uncertainties in the parameters occurring in the calculations predicting the RDDR or RGDR or based on expert judgment.

*Chemical-specific toxicokinetic or toxicodynamic differences.* Test animals and humans differ not only generically (e.g., in body size) but also in compound-specific toxicokinetics or toxicodynamics. Although on average across chemicals, the DAF is intended to neither under- nor overestimate the interspecies differences, the actual interspecies difference for any particular chemical is unknown in the absence of chemical-specific data. This uncertainty is addressed by subsequently dividing by a distribution for animal-to-human uncertainty (AHU), reflecting the additional differences in sensitivity between animal and median human beyond those addressed by the DAF (i.e., the toxicokinetic/dynamic differences specifically related to the chemical considered). For instance, assuming a log-normal uncertainty, one could define

*AHU* = exp(ε*_AHU_*), [7]

where ε*_AHU_* is normally distributed with a standard deviation of σ*_AHU_*. With chemical-specific toxicokinetic or toxicodynamic data, a CSAF or DDEF may be developed, resulting in

*AHU* = (CSAF or DDEF) × exp(ε*_AHU_*), [8]

where the standard deviation of ε*_AHU_* would normally be smaller than that of the default value related to Equation 6, as discussed in Supplemental Material, “Chemical-specific/data-derived toxicokinetics or toxicodynamics.”

*Additional study-specific adjustments.* Depending on the situation (e.g., experimental setup of a critical study, toxicity database), additional issues may need to be addressed to infer the equipotent dose in the median human under the target conditions. Those additional adjustments and their associated uncertainties that are specific to the study (or endpoint) are addressed in step 2 as well. The purpose of these adjustments is to account for the “other uncertainties” (OU) in characterizing the uncertainty distribution for the median human dose associated with a specified magnitude of effect, based on a specified study and endpoint. Examples of additional uncertainties include the following:

The human hazard to be assessed relates to a different duration of exposure than that in the study. For instance, when the effect was in a subchronic rather than chronic study, the animal dose for the selected magnitude of effect might have been smaller in a chronic study. Based on historical data, one can estimate the empirical distribution for the ratio of chronic to subchronic dose (e.g., using equipotent doses from studies of both durations across many chemicals). Or, in specific situations a dose–time relationship (e.g., cumulative dose = constant, analogous to Haber’s law) could be postulated, along with a distribution reflecting the uncertainty in how accurately the relationship holds.
The human hazard to be assessed relates to a different route of exposure than that in the study, such as inhalation versus oral. Again, both an empirical (e.g., ratio of inhalation to oral equipotent doses), theoretical (e.g., based on total intake or absorbed dose), or model-based [e.g., physiologically based pharmacokinetic (PBPK) model] approach can be used, along with a distribution reflecting the uncertainty in how accurately the assumed relationship is believed to hold.
The hazard is being assessed for a different exposure pattern than that in the study, such as continuous exposure in humans versus daily bolus exposure in the test animal. In this case, it is common to make assumptions about the dose–time relationship, such as peak or cumulative dose, as the basis for adjustment. If multiple assumptions are plausible, the uncertainty among the different options can be characterized through a distribution. For instance, when there is uncertainty whether a given peak exposure would be equivalent to a three times lower or a three times higher equivalent continuous dose as compared with Haber’s rule, this could be reflected by taking those values as the lower 5th and upper 95th percentiles of the equivalent dose distribution for constant exposure.

Note that in this step uncertainties are are with respect to the same magnitude of the same effect (endpoint). Uncertainties with respect to possibly different effects due to missing studies, even if they are at a similar level of severity, are not addressed here. This additional uncertainty is best addressed after completing steps 1–3, which are all related to the specific effect under consideration. For a discussion of some of these additional uncertainties, see Supplemental Material, “Cross-study/endpoint uncertainties.”

Step 3: Accounting for human interindividual variability in sensitivity. The aim of this step is to take into account differences in sensitivity across individuals in the population. For an exposure limit, for example, the result would be the uncertainty distribution for the dose associated with a specified endpoint and magnitude of effect (M*) for a “sensitive” individual, defined in terms of a percentile or incidence in the population (I*). To make these inferences, a population distribution representing the variation in equipotent doses among individuals needs to be specified. Because there are usually limited data as to the magnitude of this variation, this uncertainty needs to be taken into account as well.

Assuming a log-normal distribution for human variability, with standard deviation σ*_H_* on a log-scale, the relationship between M*, the incidence of effects I_≥M*_, and human dose *HD* is given by

I_≥M*_(*HD*) = Φ[{ln *HD* – ln *HD*(0.5_≥M*_)}/σ*_H_*], [9]

where Φ is the standard normal cumulative distribution. A similar relationship can be derived for any other assumed human variability distribution. For an exposure limit, one selects a target incidence value I*_≥M*_ and solves for dose *D*. Given that the median of the distribution *HD*(0.5_≥M*_) was calculated in step 2, this can be calculated by multiplying the median by the ratio between the I* quantile of the variability distribution and its median, denoted the human variability factor HV_I*_. For a log-normal distribution

HV_I__*_ = exp{z_I*_ σ*_H_*}, [10]

where z_I*_ is the normal *z*-score corresponding to a quantile I*_≥M*_. For instance, at a 5% incidence, *z*_5%_ = –1.64; at a 1% incidence, *z*_1%_ = –2.33. Combining Equations 2 and 10, the resulting equation is

*HD*(I*_≥M__*_) = AD_M*_ × *DAF* × HV_I__*_/(*AHU* × *OU*). [11]

For discussion of recent analyses of human variability data, see IPCS (2014). For instance, Hattis and colleagues (Hattis et al. 2002; Hattis and Lynch 2007) estimated equipotent doses in a number of individuals, and calculated the standard deviations σ*_H_* of the log-transformed equipotent doses, representing the variability in sensitivity among individuals. Then, they fitted a log-normal distribution to these standard deviations established for different chemicals (studies). They separated the available data into toxicokinetic and toxicodynamic factors, and estimated the uncertainty in the overall human variability as a combination of toxicokinetic and toxicodynamic variability. In this way, a default uncertainty distribution for intraspecies variation may be defined (IPCS 2014).

For some effects, we might suspect larger differences in sensitivity than others, or it might be known that the particular target subpopulation is highly sensitive for the agent considered. Or, we might be more uncertain for some effects than for others, for instance, for effects that did not occur in the database underlying the default distribution. In such cases, one may decide to deviate from the default distribution in the appropriate direction. If compound- and endpoint-specific toxicokinetic or toxicodynamic data are available, these may be used to define a case-specific human variability distribution, with case-specific uncertainty about that distribution (see Supplemental Material, “Chemical-specific/data-derived toxicokinetics or toxicodynamics”).

MC (Monte Carlo) calculation of *HD_M_^I^*_._ Keeping variability and uncertainty distinct in the calculation of *HD_M_^I^* requires a hierarchical approach to implementation. In addition, because the individual distributions cannot be combined in closed-form (particularly incorporating uncertainty in the extent of human variability), an MC simulation approach is necessary (see IPCS 2014, for an “approximate probabilistic approach” that can be implemented in a spreadsheet without MC simulation). Specifically, at each MC iteration, all the steps addressing uncertainty are done first, followed by the steps evaluating variability:

Evaluating uncertainty: Simultaneously draw MC samples [*j*] from AD_M__*_, *DAF*, *AHU*, *OU*, and σ*_H_*. Obtaining MC samples from AD_M__*_ is not a standard output from U.S. Environmental Protection Agency (EPA) Benchmark Dose Software (BMDS) (U.S. EPA 2014a), but can be generated with PROAST using the bootstrap method (RIVM 2012). Bayesian methods offer another approach to generating such samples, and software such as WinBUGS (version 1.4.3; Lunn et al. 2000), JAGS (version 4.0.0; Plummer 2003), or Stan (version 2.8.0; Stan Development Team 2015) can be used.
Evaluating variability: Combine (AD_M__*_[*j*] × *DAF*[*j*])/(*AHU*[*j*] × *OU*[*j*]) to obtain one sample of the “median” human dose *HD*(0.5_≥M*_)[*j*]. Next, given the target incidence I*, evaluate one sample of the human variability factor HV_I*_[*j*] = exp{z_I*_ σ*_H_*[*j*]}. Combining these results is one MC sample of the human target dose associated with a particular incidence I* and magnitude of effect M*: HD_M*_^I*^[*j*] = *HD*(0.5_≥M*_)[*j*] × HV_I*_[*j*].
The result after many samples is the uncertainty distribution for *HD_M_^I^*.

For the illustrative datasets discussed below, this procedure was used with 10^7^ MC samples for uncertainty (*j*) (for AD_M__*_, 10^3^ bootstrap samples were resampled with replacement). Datasets and computer code are available in Supplemental Material, Table S1). An example of this approach for an exposure limit was provided by van der Voet and Slob (2007) who used the term “*ICED*” (individual critical effect dose) rather than *HD*.

*Calculating population incidence for stochastic quantal endpoints*. In the deterministic interpretation of quantal endpoints, the calculated incidence directly represents the expected incidence in the overall population. However, in the stochastic interpretation, the calculated incidence relates to a single individual’s probability *M* of experiencing the quantal endpoints (such as a tumor). For this reason, the *HD_M_^I^* for stochastic and deterministic quantal endpoints cannot be directly compared. To make such comparison possible, for the stochastic interpretation, the expected incidence in the overall population needs to be calculated by integrating all possible values of *M* (Slob et al. 2014). The calculation is simplified by the preceding assumption that the underlying continuous dose–response relationships are “monotonic and parallel on a log-dose scale across species and across individuals within a species.” Specifically, let the animal dose–response function be represented by

*M_A_*(*AD*) = *f*(*AD*, θ), [12]

where *M_A_* is the magnitude of effect, *AD* is the animal dose, and *f* is the dose–response function with parameters θ. Based on “step 2,” the median human has the same magnitude of response as the animal [i.e., *M_A_* = *M_H,I_*_> 50%_] when the human dose *HD* = *AD* × *DAF*/(*AHU* × *OU*). Rearranging so that *AD* = *HD* × *AHU* × *OU/DAF*, the dose–response function for the median human will be

*M_H,I_*_> 50%_(*HD*) = *f*(*HD* × *AHU* × *OU/DAF*, θ), [13]

with the same model parameters θ. From “step 3,” the equipotent dose across human individuals is distributed log-normally with log-transformed standard deviation σ*_H_*. Therefore, the magnitude of effect for a particular percentile of the population with *z*-score *z*, will be

*M_H,z_*(*HD*) = *f*(exp[*z* × σ*_H_*] × *HD* × *AHU* × *OU* / *DAF*, θ). [14]

For a log-normally distributed population of equipotent doses, *z* has a normal distribution. Therefore, the population arithmetic mean of *M_H_* will be equal to the expected value of *M_H,z_* over a normally distributed *z*:

<*M_H_*(*HD*)> = ∫ *f*(exp[*z* × σ*_H_*] × *HD* × *AHU* × *OU* ÷ *DAF*, θ) φ(*z*) *dz*, [15]

where φ(*z*) is the standard normal probability density.

In the case of a stochastic quantal endpoint, *M_H_* is the “individual probability of effect,” which, averaged over the population in Equation 15, would be, by definition, equal to the expected population incidence of effect. Uncertainties in the quantities θ, *DAF*, *AHU*, *OU*, and σ*_H_* would then need to be propagated through the calculation to derive the uncertainty in this population incidence.

MC calculation of population incidence for stochastic quantal endpoints. As with the prototypical implementation of the unified probabilistic framework described above, implementing the calculation of population incidence for a stochastic quantal endpoint (Equation 1) requires an MC simulation. As was the case for calculating *HD_M_^I^*, all the steps addressing uncertainty are performed first, followed by the steps evaluating variability. In particular, at each value of human dose *HD* of interest:

Evaluating uncertainty: Simultaneously draw MC samples [*j*] from θ, *DAF*, *AHU*, *OU*, and σ*_H_*. Note that θ, which is generally multidimensional because most dose–response functions have more than one fitted parameter, has replaced the scalar (one-dimensional) quantity AD_M*_ from above. Obtaining MC samples from θ is not a standard output from BMDS, but can be generated with PROAST using the bootstrap method. Bayesian methods offer another approach to generating such samples, and software such as WinBUGS, JAGS, or Stan can be used.
Evaluating variability: Generate a human population by drawing *N* samples *z*[*k*] from a standard normal distribution, and calculate the mean value over *z* of *M_H_*:

<*M_H_*(*HD*)>[*j*] = Σ*_k_*_= 1…_*_N_ f*(exp[*z*[*k*] × σ*_H_*[*j*]] × *HD* × *AHU*[*j*] × *OU*[*j*]/*DAF*[*j*], θ[*j*])/*N*, [16]

where *N* is large enough for convergence.

The result after many samples [*j*] is the uncertainty distribution for <*M_H_*(*HD*)>. For a stochastic quantal endpoint, this equals the expected population incidence of the quantal effect. This procedure was used for the stochastic quantal treatment of tumors with 10^7^ MC samples for uncertainty (*j*) (for θ, 10^3^ bootstrap samples were resampled with replacement) and 10^4^ MC samples for variability (*k*). Datasets and computer code are available in Supplemental Material, Table S1.

*Illustrative datasets analyzed.* We used two datasets contained as examples in the PROAST software (RIVM 2012) to illustrate the approach: BW changes in rats and forestomach tumors in male mice. The tumor dataset is analyzed multiple ways—as deterministic quantal data, and as stochastic quantal data, and with extra risk levels of 10% and 1%. As discussed above, the *HD_M_^I^* outputs obtained for stochastic and deterministic quantal endpoints cannot be directly compared, so for the stochastic interpretation, the expected tumor incidence in the overall population was also calculated.

The uncertainty distributions for each step are based on the following:

The uncertainty in AD_M*_ (BMD at BMR = M*) is estimated via the bootstrap method in PROAST. To address model uncertainty, a standard set of models is fitted, with the results of all models having goodness-of-fit *p*-values > 0.05 combined with equal weight.
The distributions for *DAF* and *AHU* from Bokkers and Slob (2007), based on historical data on interspecies BMD ratios, are assumed:
*DAF* = (BW_animal_/BW_human_)^(1 – α)^, with α assumed to have a normal distribution with mean 0.7 and standard deviation 0.024.
*AHU* has a log-normal distribution with a geometric mean of 1 and a geometric standard deviation of 2.0. Notably, this distribution includes the current U.S. EPA default animal-to-human uncertainty factor (*UF_A_*) of 3 applied after application of a deterministic DAF within its 95% confidence interval (CI) (U.S. EPA 1994, 2011b).

The combined distribution for the animal-to-human adjustment, when applied to rats or mice, includes the commonly used animal-to-human factor of 10 within its 95% CI.

*OU* is omitted from the analysis (the critical study is assumed to be an adequate chronic study).
The distribution for σ*_H_* is based on a reanalysis by IPCS (2014) of published human toxicokinetic (37 datasets) and toxicodynamic (26 datasets) data compiled by Hattis and Lynch (2007). The result is a log-normal distribution for σ*_H_* with a geometric mean of 0.746 and a geometric standard deviation of 1.59. The resulting distribution for the human variability factor HV_I*_ (Equation 10), when evaluated at an incidence I* ≤ 5%, includes the commonly used human variability factor of 10 within its 95% CI.

## Results

**Figure 4:**
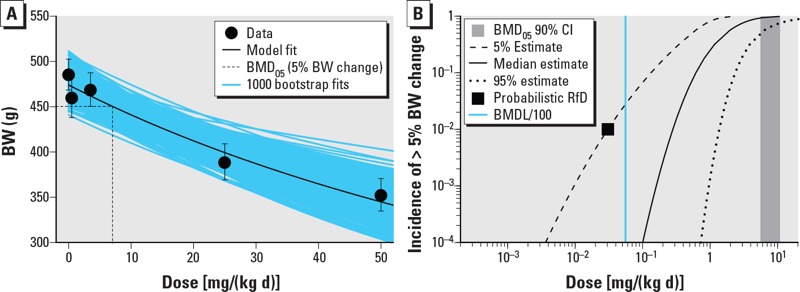
Results of analysis of example continuous dataset [rat body weight (BW) changes] as a function of dose (milligrams per kilogram BW per day). (*A*) Representative benchmark dose (BMD) modeling results using the Hill model with M* = 5% change. (*B*) Median estimate and 5th and 95th percentile estimates for the incidence (*I*) of effects of size > M* (i.e., 5% change in BW) as a function of population exposure [dose; i.e, I_≥M__*_(Dose)]. For reference, also shown are the probabilistic RfDs corresponding to a 1% incidence of effects of size > M* at 95% (one-sided) confidence (black square), the 90% (two-sided) CI for the benchmark dose (vertical gray shaded area), and a deterministic RfD equal to the BMDL/100 (vertical blue line).

**Figure 5:**
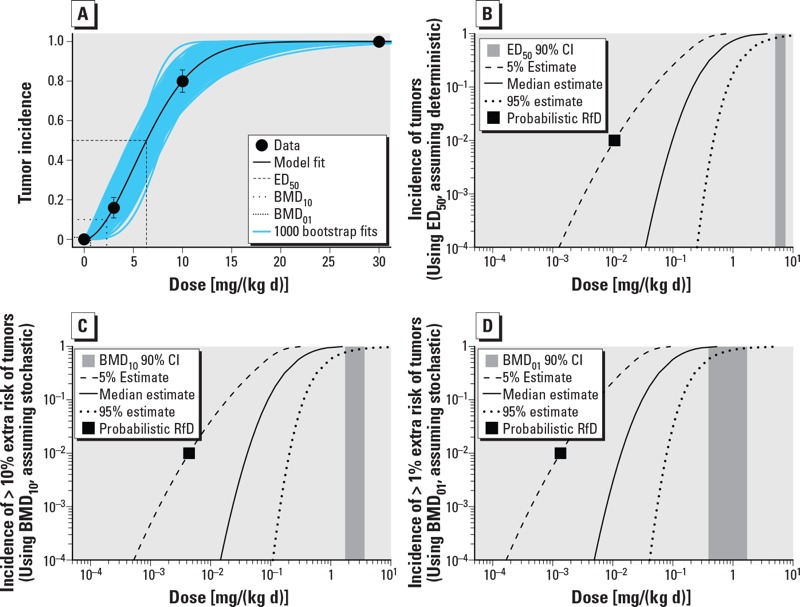
Results of analysis of example quantal dataset (forestomach tumors in mice) as a function of dose (milligrams per kilogram BW per day). (*A*) Representative benchmark dose (BMD) modeling results using the Weibull model. Multiple BMD estimates are shown, with the ED_50_ corresponding to M* = tumor, and the BMD_10_ and BMD_01_ corresponding to M* = 10% and 1% extra risk, respectively. (*B–D*) Median estimate and 5th and 95th percentile estimates ) for the incidence (*I*) of effects of size > M* as a function of population exposure [dose; i.e, I_≥M__*_(Dose)]. In (*B*), mouse forestomach tumors are treated as a deterministic quantal endpoint, whereas in (*C*,*D*), tumors are treated as a stochastic quantal endpoint [in (*C*), M* = 10% extra risk; in (*D*), M* = 1% extra risk)]. For reference, also shown in each panel are the probabilistic RfDs corresponding to a 1% incidence of effects of size > M* at 95% (one-sided) confidence (black square) and the 90% (two-sided) confidence interval (CI) for the benchmark dose (vertical gray shaded area).

For each example dataset, the results of each step in the probabilistic approach are summarized in Table 4: *a*) BMD modeling to estimate the animal dose–response relationship (Figure 4A and 5A), *b*) probabilistic interspecies adjustments to estimate the equipotent doses in median humans (*HD_M_*), and *c*) probabilistic estimates of human variability to estimate the equipotent dose in sensitive humans (*HD_M_^I^*) (Figures 4B and 5B–D).

**Table 4:** Summary of example probabilistic analyses.

Example	A	B	C	D
Dataset	Body weight in rats^*a*^	Forestomach tumors in mice^*b*^	Forestomach tumors in mice^*b*^	Forestomach tumors in mice^*b*^
Type of endpoint	Continuous	Deterministic quantal	Stochastic quantal	Stochastic quantal
*M* (effect metric)	Percent change in body weight	Tumor/no tumor	Individual probability (extra risk) of tumor	Individual probability (extra risk) of tumor
M* (critical effect size)	5%	Tumor^*c*^	10% extra risk	1% extra risk
*AD*_M*_ [critical ED (BMD) in chronic animal study]	(5.6, 11)	(5.1, 7.5)	(1.7, 3.7)	(0.39, 1.72)
*HD*_M__*_ (equipotent dose in median human)	(0.53, 5.5)	(0.19, 1.9)	(0.076, 0.83)	(0.021, 0.33)
I* (target incidence protected)	1%	1%	1%	1%
*HD*_M*_^I*^ (equipotent dose in sensitive human)^*d*^	(**0.031**, 1.4)	(**0.011**, 0.47)	(**0.0044**, 0.21)	(**0.0013**, 0.079)
The numbers in parentheses represent the 5th and 95th percentiles, respectively, of the derived uncertainty distributions. All numbers representing dose are in mg/kg BW day. Values are rounded to two significant figures. ^***a***^Use control BW of 0.496 kg for DAF. ^***b***^Use standard BW of 0.03 kg for DAF. ^***c***^For the deterministic quantal treatment of tumors, BMD analysis uses ED_50_. ^***d***^“Sensitive human” is defined by the target incidence, here 1%; an exposure limit (“probabilistic RfD”) can be based on the 5th percentile of the derived uncertainty distribution of *HD*_M*_^I*^ (bold), equivalent to a lower (one-sided) 95% confidence limit.				

Representative BMD modeling results are shown in Figure 4A for the continuous dataset (body weight changes) and Figure 5A for the quantal dataset (tumors). For the BW changes, the exponential and Hill models were fitted, both of which had goodness-of-fit *p*-values > 0.05. For tumors, the multistage, Weibull, log-logistic, log-probit, gamma, and logistic models were fitted, of which only the multistage model failed to have a goodness-of-fit *p*-value > 0.05. These datasets show clear dose responses, and the uncertainty in the BMD is relatively modest, with CIs (ratio of 95th percentile to the 5th percentile) ranging from 1.5- to 4.4-fold.

With respect to *HD_M_*, the CIs are wider due to the additional uncertainty in the interspecies adjustment (e.g., for example A in Table 4, 5.5/0.53 = 10 vs. 11/5.6 = 2.0). In addition, the 95th percentile of the *HD_M_* is lower than that of the BMD due to the allometric scaling factor.

With respect to *HD_M_^I^*, the CIs are wider still, due to the additional uncertainty in intraspecies variability, and span a 40- to 60-fold range. The 5th percentile of the *HD_M_^I^* (Table 4, Figures 4B and 5B–D) might be used as the “probabilistic RfD,” interpreted as the lower (one-sided) 95% confidence limit on the dose at which an incidence (I*) of 1% of the population experiences effects greater than the chosen critical effect size M*. Note that the choice of percent confidence, critical effect size M*, and the protection incidence I* are informed by risk management considerations and may depend on the specific context for the exposure limit. In the tumor example, a lower value for M* (individual tumor risk) may be chosen by risk managers, even though it relates to only 1% of the population. Usually, however, risk managers may prefer to have an estimate of the expected tumor incidence in the overall population (described below).

Figures 4B and 5B–D show the 90% CIs (i.e., 5th and 95th percentiles) for *HD_M_^I^* at difference levels of incidence *I* as a function of exposure, for a specified value of M*. Based on these CIs, different options for protection incidence (in combination with M*) might be selected for deriving an exposure limit. The advantage of the probabilistic framework is illustrated by its transparency in the output: The magnitude of effect, the fraction of the population protected, and percent confidence are all explicitly and quantitatively made visible. Moreover, uncertainties related to very small magnitudes of effect and/or very small incidences in the population can be made explicit and transparent (see Supplemental Material, “Extrapolation to magnitudes of effect below a critical effect size” and “Extrapolation to very low incidences”).

In the deterministic interpretation of the observed tumor incidence (example B in Table 4, Figure 5B), the calculated incidence directly represents the expected incidence in the overall population. However, in the stochastic interpretation (examples C and D in Table 4, Figure 5C,D), the calculated incidence relates to a single individual’s tumor probability M*. Thus, the exposure limit in the deterministic case protects the relevant fraction of the population (1 – *I*) against cancer as such, whereas the exposure limits in stochastic cases protect this fraction against the specified extra risk of cancer. For this reason, the outputs obtained from the deterministic versus the stochastic interpretation of tumor data cannot be directly compared.

**Figure 6:**
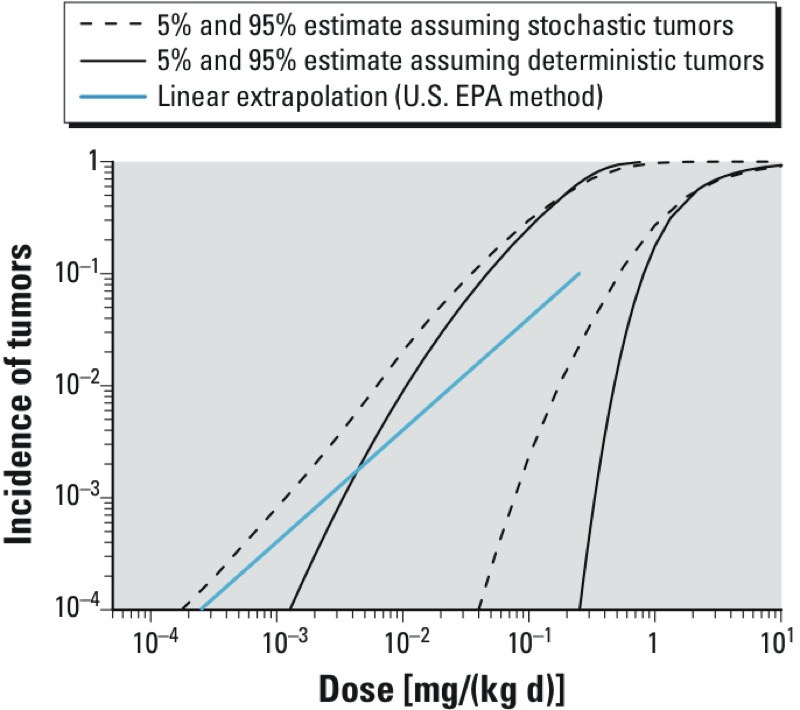
Comparison of estimated human population tumor incidences as a function of exposure [dose (milligrams per kilogram BW per day)] when treating tumors as a deterministic or a stochastic endpoint. Shown are the 90% (two-sided) CIs for human population tumor incidence calculated from the probabilistic approach, depending on whether tumors in the example dataset are treated as deterministic or stochastic quantal endpoints. For reference, also shown is the population tumor incidence derived using the default U.S. EPA method of linear extrapolation from a point of departure equal to the animal BMDL_10_ allometrically scaled by multiplying by (BW_animal_/BW_human_)^0.25^ (blue line).

As discussed in “Methods,” to compare the results from both interpretations, the expected tumor incidence in the overall population needs to be calculated for the stochastic interpretation by integrating all the incidences *I* over all possible values of *M*. The results of this analysis, including uncertainty, are shown in Table 5 and Figure 6, where the CIs on the population incidence of tumors are compared between the assumptions that tumors are “stochastic quantal” versus “deterministic quantal” effects. Results from a traditional linear extrapolation approach are also calculated for comparison.

**Table 5:** Human dose at various specified tumor incidences estimated by linear extrapolation and by the probabilistic approach based on treating tumors as deterministic quantal versus stochastic quantal effects for the example tumor dataset.

Population tumor incidence (tumor dataset)	Linear extrapolation from allometrically scaled BMDL^*a*^ (mg/kg/day)	Human dose assuming deterministic quantal effect [5th and 95th percentiles (mg/kg/day)]	Human dose assuming stochastic quantal effect [5th and 95th percentiles) (mg/kg/day)]
5%	0.11	(0.029, 0.67)	(0.020, 0.37)
1%	0.022	(0.011, 0.47)	(0.0062, 0.17)
0.1%	0.0022	(0.0034, 0.33)	(0.0012, 0.078)
0.01%	0.00022	(0.0013, 0.25)	(0.00018, 0.040)
^***a***^Based on U.S. EPA (2005) default approach, where the point of departure is the lower (one-sided) 95% confidence limit on the benchmark dose at a 10% extra risk, scaled to a human equivalent by multiplying by (BW_animal_/BW_human_)^0.25^.			

These results clearly show that the CIs for each of the two probabilistic approaches are wider than the difference between the CIs (Figure 6, Table 5). Therefore, at least in this example, the uncertainty in treating tumors as a deterministic versus a stochastic endpoint is not as great as the other uncertainties that have been characterized. Further, in this example, the result from a traditional linear extrapolation approach is not lower than the lower (one-sided) 95% confidence limit, so in that sense it is not necessarily “conservative” at the 95% level. The latter result was also found in various example cases examined by Slob et al. (2014).

## Conclusions

Compared with previous probabilistic approaches to dose–response assessment (Baird et al. 1996; Evans et al. 2001; Gaylor and Kodell 2000; Hattis et al. 2002; Slob and Pieters 1998; Swartout et al. 1998), the framework proposed here is the first to unify across the various types of endpoints that may occur in toxicological studies, such as continuous versus quantal endpoints, or cancer versus non-cancer endpoints. It does so by treating all endpoints as having a (direct or underlying) continuous response (at the level of an individual). It thereby fulfills the NRC (2009) suggestion to develop a unified approach to dose–response assessment for all endpoints. Furthermore, as discussed in Supplemental Material, the framework described here can incorporate other advances in toxicology and risk assessment, such as probabilistic exposure assessment (see Supplemental Material, “Integrating with probabilistic exposure assessment”), CSAFs or DDEFs (see Supplemental Material, “Chemical-specific/data-derived toxicokinetics or toxicodynamics”), and adverse outcome pathways (see Supplemental Material, “Extrapolation to downstream health endpoints and adverse outcome pathways” and Figure S1).

The main idea of the framework proposed here is to quantify all relevant uncertainties by distributions instead of using conservative (single) values. However, for some uncertainties, it is currently unclear how to quantify them. Importantly, the uncertainty associated with the identification of the critical studies and endpoints is difficult to quantify, and the usual (deterministic) approach of focusing on the most sensitive studies and endpoints is hard to avoid. Consequently, even if the probabilistic approach described here is implemented, the result might be more conservative than it appears. For instance, if the particular species, strain, and sex of animal were idiosyncratic (the effect would not occur in humans) or particularly sensitive compared with humans, the estimated *HD_M_^I^* would be biased downward. Furthermore, the most sensitive study from a large collection of studies will likely be more “conservative” than the most sensitive study from a smaller number of studies. The current approach remains unsatisfactory—be it in a deterministic or in a probabilistic assessment. In the short-term, the uncertainties related to the choice of the biological model might be better characterized by carrying forth multiple species/strains/sexes and endpoints to dose–response analysis (e.g., as recommended by NRC 2011), resulting in multiple *HD_M_^I^* estimates that reflect uncertainty in the chosen biological model. Furthermore, the emergence of studies using multistrain rodent panels or genetically diverse population-based rodent models (as opposed to single homogeneous, inbred strains) might provide a means to partially address these uncertainties quantitatively (e.g., Chiu et al. 2014; Rusyn et al. 2010).

In addition, even conditional on the appropriate biological model, a number of implementation challenges remain. However, although these issues have become more apparent in developing the probabilistic framework, they are equally relevant for any (deterministic) dose–response assessment method. The most important conceptual issue that has not yet been resolved is the question of which quantal endpoints should be treated as deterministic or stochastic quantal endpoints. Although for histopathological quantal data the deterministic interpretation is obvious from first principles, it is not directly clear whether cancer or malformation quantal data should be treated as stochastic or as deterministic quantal data (Slob et al. 2014). The problem is that it is not possible to directly establish this distinction from interpretation of single experiments, so additional research is needed as to what methods or datasets can distinguish between these options.

As a practical matter, there may be a tendency to treat more severe endpoints (such as tumor incidence) as stochastic because, at first sight, they seem to lead to more conservative results (although this may not always be the case). If, however, an endpoint is in reality deterministic rather than stochastic, then the outcome from the probabilistic dose–response assessment would be based on experimental error rather than biological phenomena. We repeat that this problem would not be specific for the probabilistic framework, but it equally holds for traditional deterministic dose–response assessments, such as those that apply linear extrapolation.

Another conceptual issue related to stochastic quantal endpoints concerns the definition of a toxicologically equivalent effect metric for individual probability of effect (e.g., of malformations or cancer). Specifically, it remains unclear how individual probability of effect observed in animals can be made equivalent to individual probability of effect in humans in situations where background risks differ greatly between test animals and humans. Correction for background risk can be done in various ways, such as additional, extra, or relative risk, but there are no conclusive scientific arguments to favor one over the other.

Although the framework discussed here aims to estimate health effects in the human population in terms of both *M* and *I*, it is often practical to choose a specific value of *M* (or maybe several) to simplify the calculations, as well as the output. Therefore, the choice of the critical effect size M* (or BMR) is often relevant. For continuous endpoints, current conventions as to the critical effect size M* are based on a combination of biological considerations and statistical limitations of typical dose–response data (e.g., EFSA 2009). For instance, it was argued by the European Food Safety Authority (EFSA 2009) that the transition from NOAEL to BMDL should not result in a systematic change in derived exposure limits in the long run, resulting in a recommended default for continuous endpoints of BMR = 5%. Of course, deviations in the default are allowed if biologically substantiated (e.g., BMR of ≥ 20% for liver enzyme levels, BMR of 10% for cholinesterase activity). Furthermore, one is reminded that the final output from the dose–response assessment includes the value of *M*, so that it remains visible. Consequently, one might consider requiring a lower value for *I* if the value of *M* is suspected to be higher than desirable from a public health perspective. For deterministic quantal endpoints, the value of M* is implicitly defined by the data (i.e., the associated severity category), although in some cases more than one category may be reported (e.g., “mild,” “moderate,” “severe”). For stochastic (quantal) endpoints, M* relates to the individual probability of effect (although in this case, the overall population incidence can be calculated as well, in which case *M* vanishes).

In addition, there is of course the issue of choosing values (i.e., uncertainty distributions) to be used as inputs in the probabilistic dose–response assessment. First, it should be noted that the uncertainty in the BMD is quantified by the BMD CI. In the probabilistic framework, this uncertainty directly propagates through to the overall uncertainty in the outcome of the dose–response assessment. In this way, it is directly visible to what extent designing more quantitatively informative experiments would improve a specific dose–response assessment, that is, it might indicate that further improvement would substantially decrease the overall uncertainty in the *HD_M_^I^*, or that the impact would be minor. In terms of the adjustments from the POD, uncertainty distributions for particular aspects have been suggested based on meta-analyses of historical data (Bokkers and Slob 2005, 2007; Hattis et al. 2002; Hattis and Lynch 2007), and reviewed by the IPCS (2014). Thus, in those cases where no case-specific information for a given aspect is available, these distributions may be applied as a preliminary “default” distribution in probabilistic dose–response assessments. The historical data underlying these distributions were not generated for that purpose, and it might be argued that they are not always perfectly representative or highly informative. The fact that the probabilistic methodology exists makes it highly valuable to gather and/or generate data that may lead to better-supported uncertainty distributions. Therefore, further research and exploration of historical data that may inform the uncertainty distributions would be highly useful. One of the greatest challenges is a better characterization of human toxicodynamic variability, for which there are much fewer data than for toxicokinetic variability. Emerging molecular-biology and high-throughput systems, such as use of genetically diverse populations of human cells, offer some opportunities to address this data need in a more expedited fashion (Abdo et al. 2015; Zeise et al. 2013).

Furthermore, we note that issues in choosing input values hold equally for nonprobabilistic dose–response assessments; the main difference is that the latter methods often use single default values, most of which have been generally accepted by the risk assessment community, largely by convention. However, the current single default values lead to point estimates with unknown levels of confidence and unspecified levels of the protection of the population. By contrast, the probabilistic framework allows one to examine quantitatively the uncertainty, variability, and magnitude of effect associated with dose–response assessments using such conventional approaches. In the examples provided here using the postulated uncertainty distributions, the result of default approaches, such as dividing an animal BMDL by 100 or linearly extrapolating from an allometrically scaled animal BMDL, were higher than the (one-sided) 95% confidence limit of the probabilistic outputs for the protection goals, in terms of the magnitude of effect and population incidences illustrated. A similar result was found in case studies of carcinogens by Slob et al. (2014) when comparing a probabilistic calculation with linear extrapolation. Our results imply that these traditional deterministic approaches are not necessarily conservative in the sense that the derived “virtually safe” dose does not always reach 95% confidence.

Ultimately, as noted by the NRC (1994, 2009), a probabilistic framework will provide a substantially more complete quantitative characterization of hazard. In particular, in conjunction with exposure data, the relative impact of different risk management options—in terms of magnitude of effect, incidence in the population, and degree of confidence—will be much more explicit and transparent. We envision that this will lead to better-informed risk management decisions.

## Supplemental Material

(293 KB) PDF

(219 KB) ZIP
