# Supplementary material for: A Unified Probabilistic Framework for Dose–Response Assessment of Human Health Effects
Source: Environ Health Perspect. 2015 May 22;123(12):1241–54. doi: 10.1289/ehp.1409385 (PMC4671238; doi:10.1289/ehp.1409385)
Supplement: (293 KB) PDF [file ehp.1409385.s001.acco.pdf]

Environ Health Perspect
DOI: 10.1289/ehp.1409385

Note to Readers: *EHP* strives to ensure that all journal content is accessible to all readers. However, some figures and Supplemental Material published in *EHP* articles may not conform to 508 standards due to the complexity of the information being presented. If you need assistance accessing journal content, please contact ehp508@niehs.nih.gov. Our staff will work with you to assess and meet your accessibility needs within 3 working days.

Supplemental Material

A Unified Probabilistic Framework for Dose–Response Assessment of Human Health Effects

Weihsueh A. Chiu and Wout Slob

Table of Contents

Data and R Code for Illustrative Examples

Table S1. List of files provided as Supplemental Data that can be used to reproduce the illustrative example calculations.

Additional Applications and Extensions

Performing a population assessment

Chemical-specific/data-derived toxicokinetics or toxicodynamics

Extrapolation to downstream health endpoints and adverse outcome pathways

Figure S1. Individual probability of effect generated from an underlying continuous dose-response and a deterministic or stochastic relationship to the quantal effect.

Cross-study/endpoint uncertainties

Extrapolation to magnitudes of effect below a critical effect size

Extrapolation to very low incidences

Integrating with probabilistic exposure assessment

References

Data and R Code for Illustrative Examples

Table S1. List of files provided as Supplemental Data that can be used to reproduce the illustrative example calculations.

Filename(s)	Description
atra.txt<br>methyleug.m.txt	PROAST benchmark dose modeling input files ("atra.txt" used in example A and "methyleug.m.txt" used in examples B-D and in example of population incidence calculated for a stochastic quantal endpoint).
atra.E2.bootstrap.samples.dat<br>atra.H2.bootstrap.samples.dat<br>meth.g.bmr.01.bootstrap.samples.dat<br>meth.g.bmr.10.bootstrap.samples.dat<br>meth.g.bmr.50.bootstrap.samples.dat<br>meth.l.bmr.01.bootstrap.samples.dat<br>meth.l.bmr.10.bootstrap.samples.dat<br>meth.l.bmr.50.bootstrap.samples.dat<br>meth.ll.bmr.01.bootstrap.samples.dat<br>meth.ll.bmr.10.bootstrap.samples.dat<br>meth.ll.bmr.50.bootstrap.samples.dat<br>meth.lp.bmr.01.bootstrap.samples.dat<br>meth.lp.bmr.10.bootstrap.samples.dat<br>meth.lp.bmr.50.bootstrap.samples.dat<br>meth.w.bmr.01.bootstrap.samples.dat<br>meth.w.bmr.10.bootstrap.samples.dat<br>meth.w.bmr.50.bootstrap.samples.dat	Relevant results from bootstrap sampling of the benchmark dose modeling of each dataset. For "atra," the BMR was set to 5%; for "meth," the BMRs are noted in the file name.<br><br>In each file, the first column contains the bootstrap samples for AD_M (=BMD estimate) and subsequent columns contain corresponding samples of the dose-response model parameters. Model abbreviations:<br>E2 = Exponential model<br>H2 = Hill model<br>g = Gamma model<br>l = Logistic model<br>ll = Log-logistic model<br>lp = Log-probit model<br>w = Weibull model
example.calculations.A-D.R	R script for example calculations of HD_M^I (Examples A-D in Table 4)

**Filename(s)**	**Description**
example.calculations.A-D.results.txt	Summary of results output file generated from R script for example calculations of HD_M^I
example.calculations.popincidence.R	R script for example calculation of population incidence for a stochastic quantal endpoint
example.calculations.popincidence.results.txt	Summary of results output file generated from R script for example of population incidence for a stochastic quantal endpoint

Note: The R scripts and results files provide here used fewer Monte Carlo samples as compared to the calculations reported in Tables 4-5 in the main text, so there are some minor differences in some of the calculated numbers.

Additional Applications and Extensions

The probabilistic framework outlined in the main text is sufficiently flexible so that it can be applied and extended in all sorts of ways, some examples of which are provided here.

Performing a population assessment

In contrast to deriving an exposure limit, one may alternatively want to know the incidence I^* at a particular measured or predicted exposure for a selected magnitude of effect – i.e., performing a population assessment of an exposure scenario. For instance, one may want to know the incidence of more than minimal effects (indicated by a low magnitude M^*) given current human exposure HD^*. For a fixed HD^*, this can be written

$$I^* = I_{\geq M^*}(HD^*). \qquad \text{(S-1).}$$

This quantity can be calculated using equation (8) in the main text. Alternatively, one may want to know the magnitude of effect for a given fraction of the population (I^*) at a given current exposure HD^*. For a fixed HD^*, this can be written

$$M^* = M_{I^*}(HD^*). \qquad \text{(S-2).}$$

It should be noted here that M^* now indicates the effect magnitude that will *at least* be experienced in the fraction I^*, i.e., in some individuals the effect might be greater than M^*.

As an example related to equation (S-1), with a critical endpoint defined as BW change of 10% and an exposure of 0.01 mg/(kg d), one could write $I_{\geq 10\%\mathrm{BW}}$(0.01 mg/(kg d)) for the incidence at 0.01 mg/(kg d) of greater than 10% change in body weight. For equation (S-2), for a given target incidence (e.g., 1% of the population), the corresponding magnitude of effect at an exposure of 0.01 mg/(kg d) dose could be written $M_{1\%}$(0.01 mg/(kg d)). If the exposure HD^* is

not fixed, but variable and/or uncertain in the population, then a combined probabilistic assessment of exposure and hazard can be performed.

Chemical-specific/data-derived toxicokinetics or toxicodynamics

In the deterministic approach using uncertainty factors, it is common practice to split the inter- and the intraspecies factor into two sub-factors, representing toxicokinetic and toxicodynamic differences between or within species (IPCS 1994, 2005; U.S. EPA 2014). In the probabilistic framework, the distributions for interspecies differences and human variability could be split up into two sub-distributions just as well. When, for instance, information on toxicokinetic differences between the test animal and humans is available, this information could be translated into a chemical-specific (or "data-derived") distribution expressing the uncertainty in the toxicokinetic subfactor. This toxicokinetic sub-factor may be based on toxicokinetic modeling (e.g. PBPK modeling), for instance, by calculating a (best) estimate of the ratio of internal dose metrics in rat vs. human, given the same external dose. In a deterministic risk assessment this best estimate would be used, and replace the default toxicokinetic subfactor (usually around 3 or 4). However, one should also include the uncertainty in the chemical-specific estimate of that subfactor, which in some cases may be larger than in others, depending on the quality of the underlying toxicokinetic data. In a probabilistic framework this uncertainty in a chemical-specific adjustment factor (IPCS 2005) or data-derived extrapolation factor (U.S. EPA 2014) can be taken into account.

It should be noted that if a full PBPK model was used to predict internal dose metrics in both the test animal and in humans, care should be taken in using the appropriate measure of internal dose (such as area under the concentration curve or maximum concentration) based on what is known about the mode of action or adverse outcome pathway. Further, if the uncertainty

in the ratio of internal dose in human vs. animal was evaluated, there is no longer a need to apply the (uncertain) allometric correction factor, as this applies to external dose only. Size differences between animal and humans have been taken into account in the respective PBPK models by using species-specific physiological parameters. In some cases the distribution for the toxicokinetic interspecies factor could have a median value that is much greater than the default sub-factor (e.g. dioxin risk assessment based on developmental effects). But even in such cases the uncertainty in its estimate should be taken into account.

In more advanced analyses based on PBPK modeling one may distinguish between variability and uncertainty. For instance, one may assume that various physiological parameters (e.g. cardiac output, organ weights) vary among individuals, while other parameters (e.g. metabolic rate, partition coefficient) are both variable and uncertain, i.e. the assumed variability might be considered uncertain itself. Hierarchical Bayesian approaches can be used to account for both uncertainty and variability simultaneously in PBPK modeling (Bois et al. 1996; Bois et al. 2010; Chiu et al. 2009).

Extrapolation to downstream health endpoints and adverse outcome pathways

In some cases, the measure of toxicological effect *M* in humans can be related to further downstream endpoints, such as if an adverse outcome pathway can quantify the linkage between a change in effect metric for a given parameter and the likelihood of an adverse health outcome. For instance, if the measure of toxicological effect is a percent change in blood pressure, then using existing clinical and/or epidemiologic data for stroke, it may be possible to quantitatively link changes in blood pressure to changes in the risk of stroke. Similarly, to the extent that high throughput testing can provide dose-response data on relevant biological perturbations and a

quantitative adverse outcome pathway can be developed, the relationship between the magnitude of such perturbations and the likelihood of an ultimate adverse outcome could be estimated.

Therefore, if a quantitative relationship between the variable M and an "apical" endpoint E can be established, then the results of the probabilistic hazard characterization can be used to predict the individual probability of effect and/or population incidence of apical endpoints (see Supplemental Figure S1 for an illustration). In particular, if the function $P(E|M)$, the probability of apical endpoint E given a toxicological effect of magnitude M, is known, then, at a human dose $HD(0.5_{\geq M})$, the $P(E|M)$ is equal to the individual probability of E occurring for the median individual. Furthermore, given $I_{\geq M}(HD)$, the incidence of effects greater than M at HD (main text equation (8)), the population incidence of E can be calculated by integrating the product of $P(E|M^*)$ and the derivative of $I_{\geq M^*}(HD^*)$ with respect to M^*. The probabilistic framework allows for propagating uncertainties through this type of extrapolation as well.

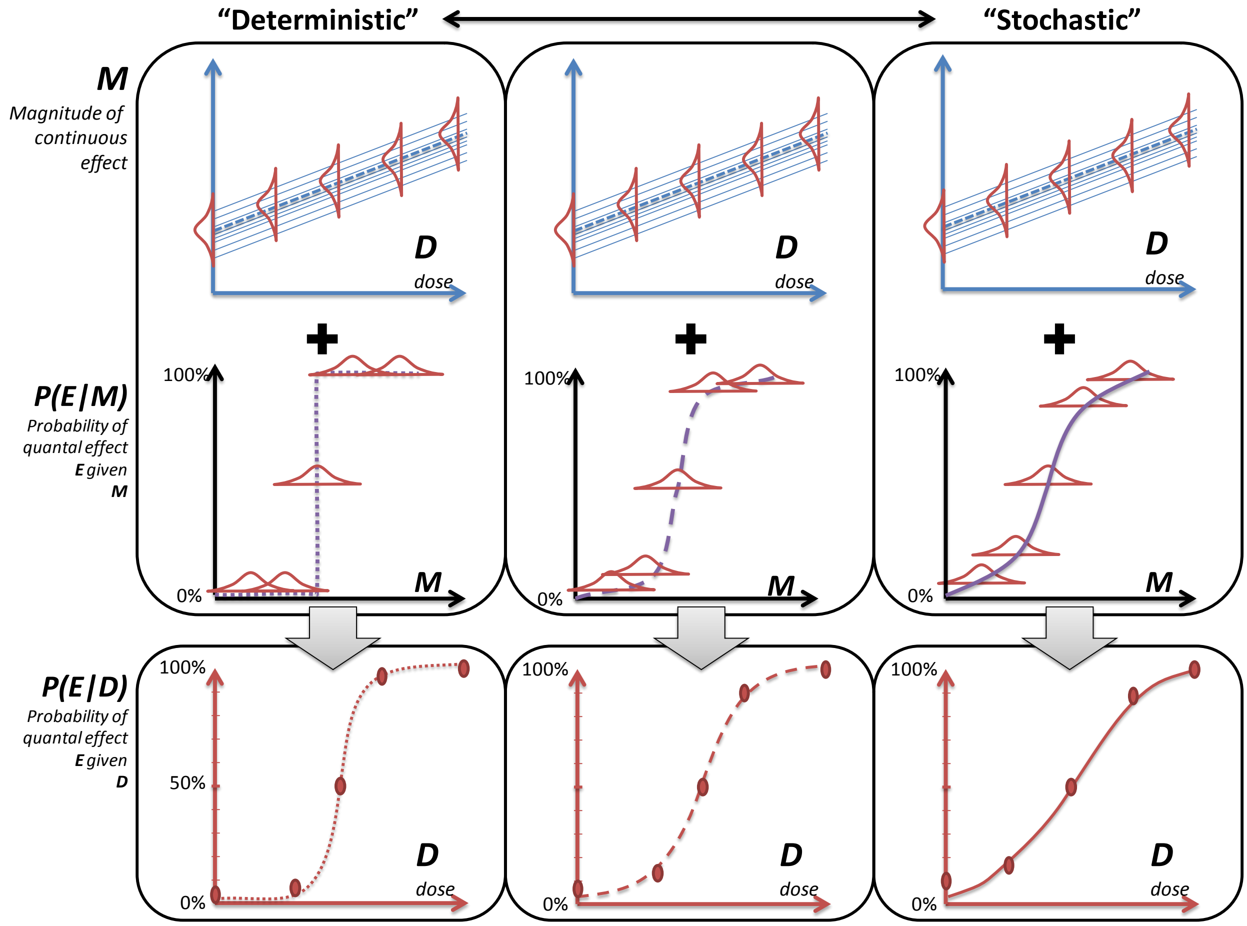

Figure S1. Individual probability of effect generated from an underlying continuous dose-response and a deterministic or stochastic relationship to the quantal effect. The left panel illustrates a "deterministic" quantal effect, analogous to Figure 1, whereas the middle and right panels illustrate "stochastic" quantal effects, analogous to Figure 2.

Cross-study/endpoint uncertainties

If there are substantial gaps in the toxicity database, then there is uncertainty in the sense that another critical effect might have been found if additional studies had been conducted. Data gaps may include missing critical species (e.g., no rat study) or missing study type (e.g., no reproductive study). In such a case, a distribution as to the ratio of the BMDs for similar levels of severity can be postulated, and it can be treated as an "additional uncertainty" similar to the study-/endpoint-specific uncertainties (***OU***) discussed above. However, to make clear that the end result is relating to a possibly different endpoint, it is recommended that this uncertainty (analogous to the "database" factor applied in deterministic analyses) be included only after the results for the specific effect have been completed. Empirical information on these types of uncertainties may be found in the usual approach by evaluating PoD ratios in chemicals for which two study types are available (e.g., Evans and Baird 1998; Janer et al. 2007a, 2007b, 2007c).

Extrapolation to magnitudes of effect below a critical effect size

Sometimes, effect sizes smaller than the critical effect size M^* that was used in the BMD analysis of the animal study may be of interest. One example is for a severe stochastic quantal effect for which the individual probability of effect M^* would not be considered "acceptable" at levels like 10% of even 1%. In such cases, it may be desirable to extrapolate to smaller effect size. Statistical modeling approaches can estimate the dose-response relationship at any effect size, though the uncertainty generally increases substantially at lower levels of M^*. Recently, modeling averaging has been proposed as a robust approach to extrapolating to low effect sizes (Wheeler and Bailer 2013). The probabilistic framework makes these uncertainties explicit and visible in the risk assessment.

Extrapolation to very low incidences

Another dimension of potential extrapolation problems is in relation to incidence. For instance, for a severe magnitude of effect, it might be desirable to restrict the incidence to a very small percentage of the population (e.g., 0.01% or even lower). The approach presented in the main text assumes a unimodal distribution for the variation in sensitivity in humans, and the width of the distribution can be estimated generically (i.e., not specific to the particular chemical and effect) based on other data (e.g., drug metabolism, human clinical studies, etc.). Even in this case, the uncertainty in human variability has a very large impact on estimates of human doses corresponding to very small incidences. Moreover, the unknown shape of the distribution in the extreme tails and the potential presence of multiple modes contribute to even greater uncertainty. In general, extrapolations to such very sensitive individuals will likely remain highly uncertain due to lack of accessibility to observation.

Integrating with probabilistic exposure assessment

A fully probabilistic risk characterization could be accomplished by combining the probabilistic hazard characterization discussed here with a probabilistic exposure assessment that accounts for both uncertainty and variability.

The "individual margin of exposure" (iMoE) is one approach that has been proposed (e.g., van der Voet et al. 2009). The procedure is the same as that outlined previously for calculating HD_M^I, but with exposure uncertainty evaluated along with hazard uncertainty, and exposure variability evaluated along with hazard variability. In particular, given a particular sample of each variability distribution (i.e., one for exposure and one for hazard), a "random individual" is drawn from each distribution. The ratio between the dose associated with the specified endpoint/magnitude of effect and the exposure constitutes the iMoE. Thus, based on a

large number of randomly drawn individuals, a single sample of the variability distribution for the iMoE is generated. This entire procedure is then repeated by sampling input values from the uncertainty distributions to characterize the uncertainty.

References

Bois FY, Gelman A, Jiang J, Maszle DR, Zeise L, Alexeef G. 1996. Population toxicokinetics of tetrachloroethylene. Arch Toxicol. 1996;70(6):347-355.

Bois FY, Jamei M, Clewell HJ. 2010. PBPK modelling of inter-individual variability in the pharmacokinetics of environmental chemicals. Toxicology. 2010 Dec 30;278(3):256-267.

Chiu WA, Okino MS, Evans MV. 2009. Characterizing uncertainty and population variability in the toxicokinetics of trichloroethylene and metabolites in mice, rats, and humans using an updated database, physiologically based pharmacokinetic (PBPK) model, and Bayesian approach. Toxicol Appl Pharmacol. 241(1):36-60.

Evans JS; Baird SJS. 1998. Accounting for Missing data in Noncancer Risk Assessment. Human and Ecological Risk Assessment. 4:291–317.

International Program on Chemical Safety (IPCS). 1994. Assessing human health risks of chemicals: Derivation of guidance values for health-based exposure limits. Geneva, World Health Organization, International Programme on Chemical Safety (Environmental Health Criteria 170). Available: http://www.inchem.org/documents/ehc/ehc/ehc170.htm [accessed 28 April 2015]

International Program on Chemical Safety (IPCS). 2005. Chemical-Specific Adjustment Factors for Interspecies Differences and Human Variability: Guidance Document for Use of Data in Dose/Concentration-Response Assessment. World Health Organization, Geneva. Available: http://whqlibdoc.who.int/publications/2005/9241546786_eng.pdf [accessed 28 April 2015]

Janer, G., Hakkert, B.C., Slob W, Vermeire, T., and Piersma, A.H. 2007a. A retrospective analysis of the two-generation study: what is the added value of the second generation? Reprod Toxicol. 24:97–102.

Janer, G., Hakkert, B.C., Piersma, A.H., Vermeire, T., and Slob W. 2007b. A retrospective analysis of the added value of the rat two-generation reproductive toxicity study versus the rat subchronic study. Reprod Toxicol. 24, 103-113

Janer G., Slob W., Hakkert B.C., Vermeire T., and Piersma A.H. 2008c. A retrospective analysis of developmental toxicity studies in rat and rabbit: What is the added value of the rabbit as an additional test species? Regul. Toxic. Pharm. 50: 206-217.

van der Voet H, van der Heijden GW, Bos PM, Bosgra S, Boon PE, Muri SD, Brüschweiler BJ. 2009. A model for probabilistic health impact assessment of exposure to food chemicals.

Food Chem Toxicol. 47(12):2926-2940.

U.S. EPA. 2014. Guidance for Applying Quantitative Data to Develop Data-Derived Extrapolation Factors (DDEF) for Interspecies and Intraspecies Extrapolation. U.S. Environmental Protection Agency, Washington, DC, EPA/100/R-14/002F. Available: http://www2.epa.gov/osa/guidance-applying-quantitative-data-develop-data-derived-extrapolation-factors-interspecies-and [accessed 28 April 2015]

Wheeler MW, Bailer AJ. 2013. An empirical comparison of low-dose extrapolation from points of departure (PoD) compared to extrapolations based upon methods that account for model uncertainty. Regul Toxicol Pharmacol. 67(1):75-82.
